# Nomogram for preoperative prediction of high-volume lymph node metastasis in the classical variant of papillary thyroid carcinoma

**DOI:** 10.3389/fsurg.2023.1106137

**Published:** 2023-02-08

**Authors:** Huahui Feng, Zheming Chen, Maohui An, Yanwei Chen, Baoding Chen

**Affiliations:** Department of Medical Ultrasound, The Affiliated Hospital of Jiangsu University, Zhenjiang, China

**Keywords:** papillary thyroid carcinoma, high-volume lymph node metastasis, risk factors, nomogram, ultrasonography

## Abstract

**Introduction:**

The objective of our study was to construct a preoperative prediction nomogram for the classical variant of papillary thyroid carcinoma (CVPTC) patients with a solitary lesion based on demographic and ultrasonographic parameters that can quantify the individual probability of high-volume (>5) lymph node metastasis (HVLNM).

**Materials and methods:**

In this study, a total of 626 patients with CVPTC from December 2017 to November 2022 were reviewed. Their demographic and ultrasonographic features at baseline were collected and analyzed using univariate and multivariate analyses. Significant factors after the multivariate analysis were incorporated into a nomogram for predicting HVLNM. A validation set from the last 6 months of the study period was conducted to evaluate the model performance.

**Results:**

Male sex, tumor size >10 mm, extrathyroidal extension (ETE), and capsular contact >50% were independent risk factors for HVLNM, whereas middle and old age were significant protective factors. The area under the curve (AUC) was 0.842 in the training and 0.875 in the validation set.

**Conclusions:**

The preoperative nomogram can help tailor the management strategy to the individual patient. Additionally, more vigilant and aggressive measures may benefit patients at risk of HVLNM.

## Introduction

The prevalence of thyroid cancer is increasing worldwide (1). Notably, papillary thyroid carcinoma accounts for 85% of differentiated thyroid cancers with a high 10-year survival rate (2–4). CVPTC is the most prevalent variant and is believed to be a less aggressive histological subtype that has a lower risk for death and metastatic disease (5). Prophylactic central lymph node dissection remains controversial according to different guidelines (6–8). However, up to 50% of papillary thyroid carcinoma (PTC) patients develop lymph node metastasis (LNM) (9). Large-volume or high-volume LNM (HVLNM) was defined as >5 metastatic lymph nodes (8). Increasing evidence has shown that patients with HVLNM have poorer outcomes than those with small-volume LNM, which includes higher recurrence rates and lower disease-free survival (10, 11). Consequently, the latest American Thyroid Association (ATA) guidelines have determined clinical N1 disease or >5 pathological lymph nodes (less than 3 cm) as characteristics of patients with an intermediate risk for postoperative risk stratification (8).

Ultrasound (US) is the primary choice for routine thyroid examination and preoperative staging of thyroid cancer. Several associations have published reporting systems for the assessment of thyroid nodules based on ultrasonographic patterns that are helpful in diagnosing nodules (8, 12–14). However, due to the complex anatomical structure of the neck and the physical limitations of US, the diagnostic performance of preoperative US evaluation in detecting lymph node involvement in the cervical region, especially in the central compartment, is not particularly effective (15). Another reason that the detection rates of positive lymph nodes are low is that the procedure relies heavily on the proficiency of operators. Therefore, during the preoperative examination, the potential risks of LNM and HVLNM may be overlooked, further misleading the management of vulnerable patients.

Many studies have focused on the association between imaging patterns of thyroid nodules and LNM. A few studies have concentrated on the risk factors for HVLNM (16–20). Furthermore, in these studies, multifocal lesions and postoperative diagnosis were analyzed in most cases. And the association between preoperative ultrasonographic features and HVLNM, such as capsule morphology, has never been investigated thoroughly. Clinical decisions may be altered if feasible preoperative prediction models can be established, and more aggressive treatment modalities may be found suitable for some CVPTC patients.

Thus, this study aimed to construct a preoperative nomogram to predict HVLNM in CVPTC patients with a solitary lesion based on demographic and ultrasonographic features. Besides, the predictive value of the nomogram was assessed using a validation set consisting of the patients from the last 6 months of the cohort. The developed nomogram may help clinicians select a follow-up approach for the entire diagnosis and treatment process.

## Materials and methods

### Patient selection

**Figure 1:**
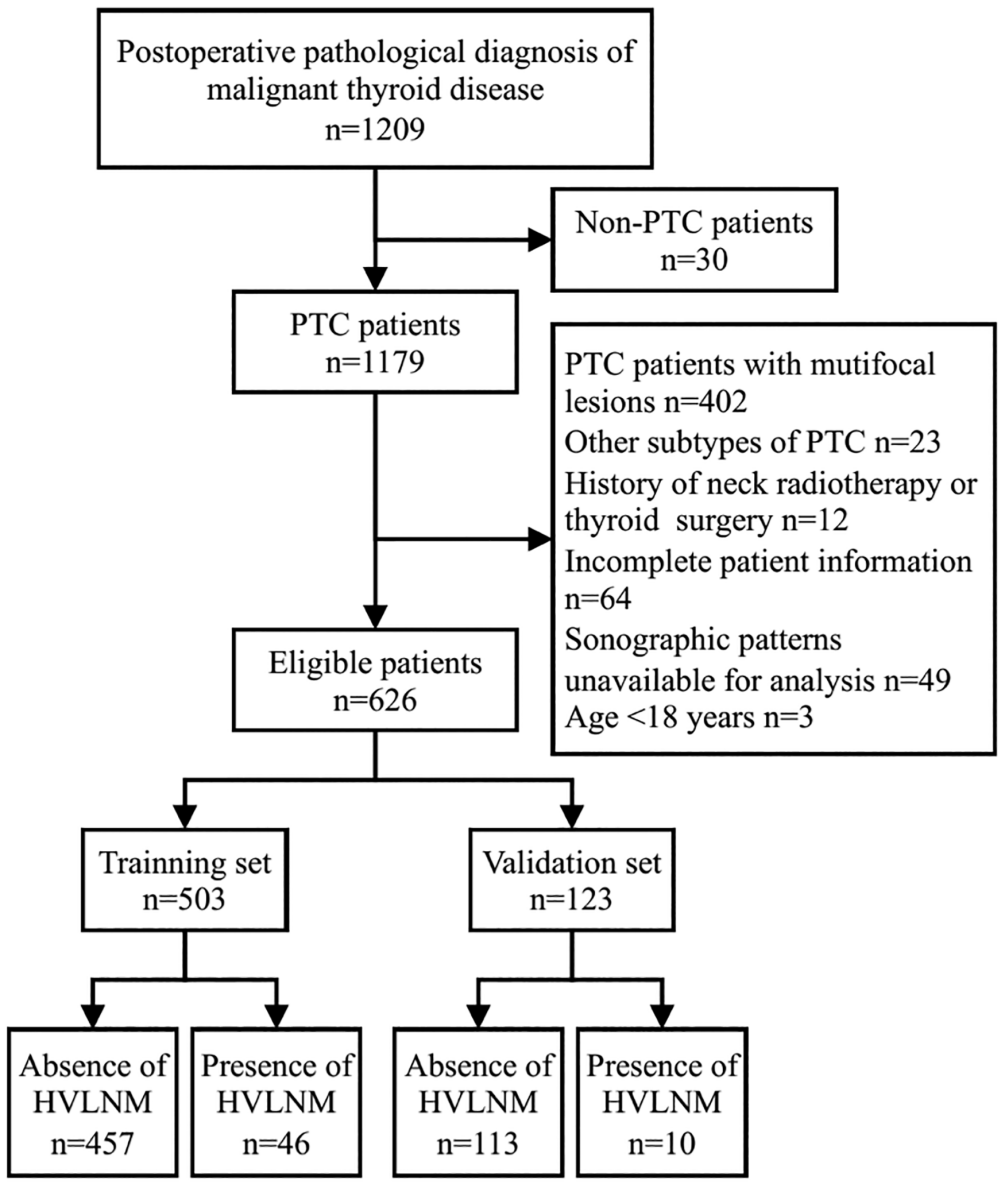
The flowchart of the patient selection process.

This retrospective study was approved by the Ethics Committee of the Affiliated Hospital of Jiangsu University, and the requirement for written informed consent was waived. The records of 626 patients diagnosed with PTC between December 2017 and November 2022 were retrospectively assessed. These medical records were reviewed to collect data, including sex, age, final pathological diagnoses, and preoperative ultrasonographic findings. The inclusion criteria were as follows: (1) postoperative pathological diagnosis of CVPTC, (2) age ≥18 years, and (3) complete preoperative thyroid US. The exclusion criteria were as follows: (1) history of neck radiotherapy or thyroid surgery, (2) incomplete patient information in the hospital database, and (3) sonographic patterns unavailable for analysis. Preoperative fine-needle aspiration (FNA) and US were performed to eliminate suspicious lesions in the unresected thyroid tissue. Prophylactic central lymph node dissection (CLND) was performed in all patients, whereas lateral lymph node dissection (LLND) was performed based on preoperative imaging reports and US-guided FNA biopsy results for suspicious metastatic lymph nodes. Postoperative pathology is the gold standard for lymph node metastasis. HVLNM is defined as more than 5 positive metastatic lymph nodes on postoperative pathologic diagnosis. The flowchart of the patient selection process is shown in Figure 1.

### Preoperative US examination and image analysis

Two experienced radiologists independently assessed the US patterns of the suspicious nodules. When discrepancies emerged, a senior radiologist reviewed the images. Nodules were classified according to the 2020 Chinese Thyroid Imaging Reporting and Data System (C-TIRADS) (14). Ultrasonographic characteristics were further categorized as follows: tumor size (≤10 or >10 mm), composition (mixed or solid), echogenicity (hyperechoic, isoechoic, hypoechoic, or markedly hypoechoic), shape (wider than tall or taller than wide), margin (circumscribed or not circumscribed), calcifications (absent, microcalcifications, macrocalcifications, or mixed calcifications), vascular pattern (avascularity, peripheral, mainly peripheral, mainly central, or mixed vascularity), capsular contact (no contact, ≤50% or >50%), and ETE on US (absent or present). The tumor’s size was defined by its maximum diameter on US. The tumor shape was evaluated based on the transverse dimension. Microcalcifications were defined as hyperechoic foci that were equal to or less than 1 mm in diameter. Calcifications >1 mm were classified as macrocalcifications (14). When microcalcifications and macrocalcifications presented simultaneously in a nodule, they were classified as mixed calcifications. Disruption of the thyroid capsule and gross invasion of the perithyroidal structures were defined as ETE on US. Bulging of the normal thyroid contour without capsule disruption would not be defined as ETE. The degree of capsular contact was calculated according to the proportion of the nodule perimeter in contact with the capsules on the images where the nodule was in greatest contact with the capsules (21). Vascular patterns were detected using color Doppler US.

### Statistical analysis

The entire group was divided into the training set and the validation set. Patients included from the last 6 months of the study period were used for the validation set. The developed model from the training set was then tested in the validation set. All statistical analyses were performed using SPSS version 29 and R programming language. Statistical significance was defined as *P* < 0.05. Categorical variables are presented as numbers (%). Univariate analysis was performed for categorical variables using the chi-squared test or Fisher’s exact test. Variables with a *P*-value less than 0.05 in the univariate analysis were included in the multivariate analysis to establish a logistic regression model.

A nomogram was generated using independent predictors from the multivariate analysis to visualize the individual probability of HVLNM. The model discriminatory ability was determined using the area under the receiver operating characteristic (ROC) curve (AUC), known as the concordance index. An AUC of 1 represents a perfect model, whereas an AUC of 0.5 represents a random classifier. Calibration curves were plotted to compare the predicted versus actual probabilities. Decision curve analysis (DCA) was used to estimate the net benefit.

## Results

### Clinicopathological backgrounds

In this study, 626 patients with solitary CVPTC confirmed by postoperative pathology were included between December 2017 and November 2022. 503 patients between December 2017 and May 2022 constituted the training set, and 123 patients were allocated to the validation set between June 2022 and November 2022. Patients were divided into HVLNM and non-HVLNM groups according to the number of metastatic lymph nodes confirmed by postoperative pathology. A total thyroidectomy or near-total thyroidectomy was performed in 89 patients. Thyroid lobectomy with or without the isthmus was performed in 537 patients. After surgery, 46 (9.1%) cases in the training set and 10 (8.1%) cases in the validation set had HVLNM (Table 1). Baseline demographic and ultrasonographic characteristics were summarized in Table 1. The training set showed a good agreement with the validation set, except for a difference in the presence of ETE on US (Table 1).

**Table 1:** Baseline demographic and ultrasonographic characteristics of patients with solitary CVPTC.

Characteristics		Total	Patient sets (%)		*P*-value
			Training set	Validation set	
Sex	Female	476	386 (76.7)	90 (73.2)	0.406
	Male	150	117 (23.3)	33 (26.8)	
Tumor size	≤10 mm	414	333 (66.2)	81 (65.9)	0.942
	>10 mm	212	170 (33.8)	42 (34.1)	
Composition	Mixed	51	46 (9.1)	5 (4.1)	0.065
	Solid	575	457 (90.9)	118 (95.9)	
Echogenicity	Hyperechoic	15	11 (2.2)	4 (3.3)	0.076
	Isoechoic	51	37 (7.4)	14 (11.4)	
	Hypoechoic	402	318 (63.2)	84 (68.3)	
	Markedly hypoechoic	158	137 (27.2)	21 (17.1)	
Shape	Wider than tall	189	149 (29.6)	40 (32.5)	0.530
	Taller than wide	437	354 (70.4)	83 (67.5)	
Margin	Circumscribed	22	20 (4)	2 (1.6)	0.319
	Not circumscribed	604	483 (96)	121 (98.4)	
Vascular pattern	Avascularity	266	202 (40.2)	64 (52)	0.090
	Peripheral vascularity	144	117 (23.3)	27 (22)	
	Mainly peripheral vascularity	84	75 (14.9)	9 (7.3)	
	Mainly central vascularity	49	40 (8)	9 (7.3)	
	Mixed vascularity	83	69 (13.7)	14 (11.4)	
ETE	Absent	572	472 (93.8)	100 (81.3)	<0.001
	Present	54	31 (6.2)	23 (18.7)	
Capsular contact	No contact	304	243 (48.3)	61 (49.6)	0.065
	≤50%	240	201 (40)	39 (31.7)	
	>50%	82	59 (11.7)	23 (18.7)	
Calcifications	Absent	230	182 (36.2)	48 (39)	0.366
	Microcalcifications	316	261 (51.9)	55 (44.7)	
	Macrocalcifications	43	31 (6.2)	12 (9.8)	
	Mixed calcifications	37	29 (5.8)	8 (6.5)	
C-TIRADS category	C-TIRADS 3	2	2 (0.4)	0 (0)	0.169
	C-TIRADS 4A	12	8 (1.6)	4 (3.3)	
	C-TIRADS 4B	73	58 (11.5)	15 (12.2)	
	C-TIRADS 4C	491	391 (77.7)	100 (81.3)	
	C-TIRADS 5	48	44 (8.7)	4 (3.3)	
Age	<40	217	174 (34.6)	43 (35)	0.906
	40–55	252	201 (40)	51 (41.5)	
	≥55	157	128 (25.4)	29 (23.6)	
HVLNM	Absent	570	457 (90.9)	113 (91.9)	0.724
	Present	56	46 (9.1)	10 (8.1)	

### Univariate and multivariate analyses of risk factors for HVLNM in CVPTC patients

In the training set, univariate analysis revealed that sex, age, tumor size, nodule composition and shape, ETE on US, and capsular contact were associated with HVLNM (Table 2). The incidence of HVLNM varied significantly among the three age groups. The incidences were 14.9% (26/174), 6% (12/201), and 6.2% (8/128), respectively. Moreover, capsular contact >50% presented a higher incidence than the other two groups (no contact and contact ≤50%). Tumor echogenicity, margin, calcifications, vascular pattern, and C-TIRADS category did not correlate with the presence of HVLNM.

**Table 2:** Univariate analysis of risk factors for HVLNM in the training set.

Characteristics		Non-HVLNM (*N* = 457)	HVLNM (*N* = 46)	*P*
Sex	Female	357 (92.5)	29 (7.5)	<0.05
	Male	100 (85.5)	17 (14.5)	
Age	<40	148 (85.1)	26 (14.9)	<0.01
	40–55	189 (94)	12 (6)	
	≥55	120 (93.8)	8 (6.2)	
Tumor size	≤10 mm	319 (95.8)	14 (4.2)	<0.001
	>10 mm	138 (81.2)	32 (18.8)	
Composition	Mixed	36 (78.3)	10 (21.7)	<0.01
	Solid	421 (92.1)	36 (7.9)	
Echogenicity	Hyperechoic	8 (72.7)	3 (27.3)	0.193
	Isoechoic	33 (89.2)	4 (10.8)	
	Hypoechoic	290 (91.2)	28 (8.8)	
	Markedly hypoechoic	126 (92)	11 (8)	
Shape	Wider than tall	128 (85.9)	21 (14.1)	<0.05
	Taller than wide	329 (92.9)	25 (7.1)	
Margin	Circumscribed	19 (95)	1 (5)	0.795
	Not circumscribed	438 (90.7)	45 (9.3)	
Vascular pattern	Avascularity	186 (92.1)	16 (7.9)	0.116
	Peripheral vascularity	106 (90.6)	11 (9.4)	
	Mainly peripheral vascularity	70 (93.3)	5 (6.7)	
	Mainly central vascularity	38 (95)	2 (5)	
	Mixed vascularity	57 (82.6)	12 (17.4)	
ETE	Absent	438 (92.8)	34 (7.2)	<0.001
	Present	19 (61.3)	12 (38.7)	
Capsular contact	No contact	234 (96.3)	9 (3.7)	<0.001
	≤50%	184 (91.5)	17 (8.5)	
	>50%	39 (66.1)	20 (33.9)	
Calcifications	Absent	172 (94.5)	10 (5.5)	0.128
	Microcalcifications	231 (88.5)	30 (11.5)	
	Macrocalcifications	29 (93.5)	2 (6.5)	
	Mixed calcifications	25 (86.2)	4 (13.8)	
C-TIRADS category	C-TIRADS 3	1 (50.0)	1 (50.0)	0.064
	C-TIRADS 4A	7 (87.5)	1 (12.5)	
	C-TIRADS 4B	51 (87.9)	7 (12.1)	
	C-TIRADS 4C	361 (92.3)	30 (7.7)	
	C-TIRADS 5	37 (84.1)	7 (15.9)	

The data are presented as *N* (%).

Significant factors in the univariate analysis were then included in the multivariate analysis. Multivariate logistic regression analysis demonstrated that male sex (OR 3.396, 95% CI 1.579–7.304), tumor size >10 mm (OR 2.662, 95% CI 1.156–6.132), ETE on US (OR 5.087, 95% CI 1.897–13.638), and capsular contact >50% (OR 7.377, 95% CI 2.697–20.175) were independent risk factors for HVLNM. Compared with young patients (age <40 years), middle-aged (OR 0.389, 95% CI 0.173–0.878) and older patients (OR 0.295, 95% CI 0.116–0.755) had a lower risk of HVLNM (Table 3). Nagelkerke R square for the model was 0.305.

**Table 3:** Multivariate analysis of risk factors for HVLNM.

Characteristics	OR	95% CI		*P*-value
		Lower	Upper	
Age (<40)	1	-	-	0.012
Age (40–55)	0.389	0.173	0.878	0.023
Age (≥55)	0.295	0.116	0.755	0.011
Male sex	3.396	1.579	7.304	0.002
Tumor size >10 mm	2.662	1.156	6.132	0.021
Solid composition	0.984	0.362	2.675	0.975
Taller than wide	0.956	0.443	2.060	0.908
ETE	5.087	1.897	13.638	0.001
Capsular contact (no contact)	1	-	-	<0.001
Capsular contact (≤50%)	1.738	0.694	4.356	0.238
Capsular contact (>50%)	7.377	2.697	20.175	<0.001

### Model construction and validation

**Figure 2:**
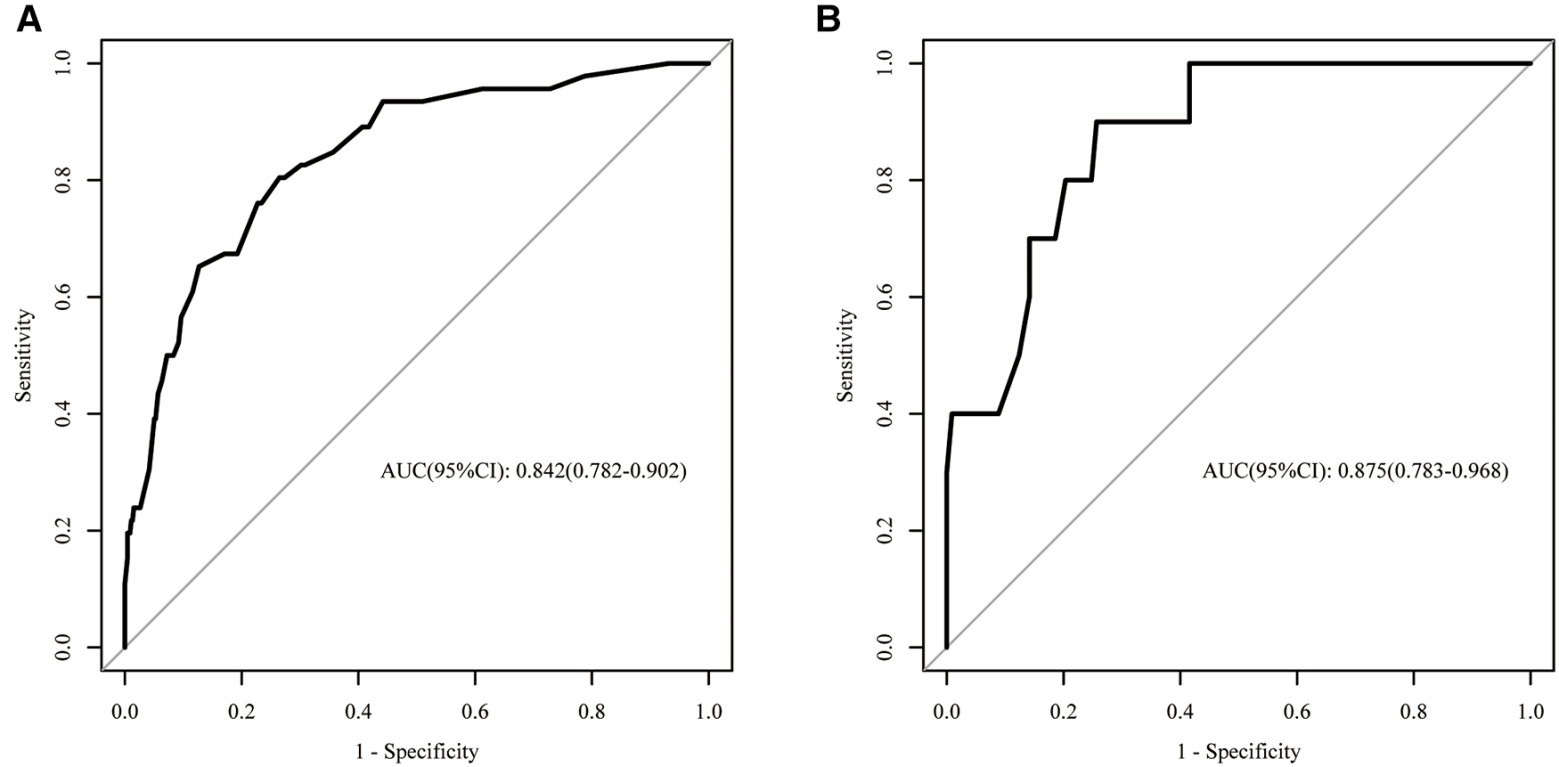
Receiver operating characteristics curves in the training set (**A**) and validation set (**B**).

The logistic regression analysis used all the independent factors to develop a predicting model. The AUC of the developed model in predicting HVLNM in the training set was 0.842 (95% CI 0.782–0.902). The sensitivity, specificity, positive predictive value, and negative predictive value of 0.804, 0.735, 0.234, and 0.974, respectively. In the validation set, the developed model acquired an AUC of 0.875 (95% CI 0.783–0.968) and yielded the sensitivity, specificity, positive predictive value, and negative predictive value of 0.900, 0.743, 0.237, and 0.988, respectively (Figure 2).

**Figure 3:**
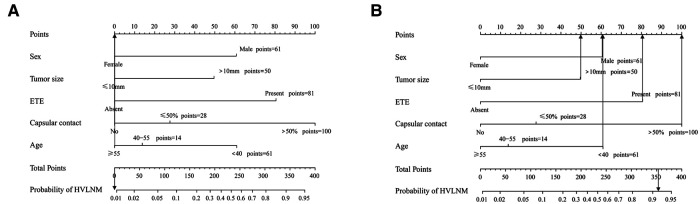
The use of the developed nomogram. (**A**) A 75-year-old lady diagnosed as non-HVLNM with a papillary thyroid microcarcinoma, extrathyroidal extension (−), capsular contact (−). The total score of this patient is 0 and the risk rate of HVLNM was <0.01. (**B**) A 37-year-old man diagnosed as HVLNM with a papillary thyroid macrocarcinoma, extrathyroidal extension (+), capsular contact (>50%). The total score of this patient is 353 and the risk rate of HVLNM was 91.6%.

**Figure 4:**
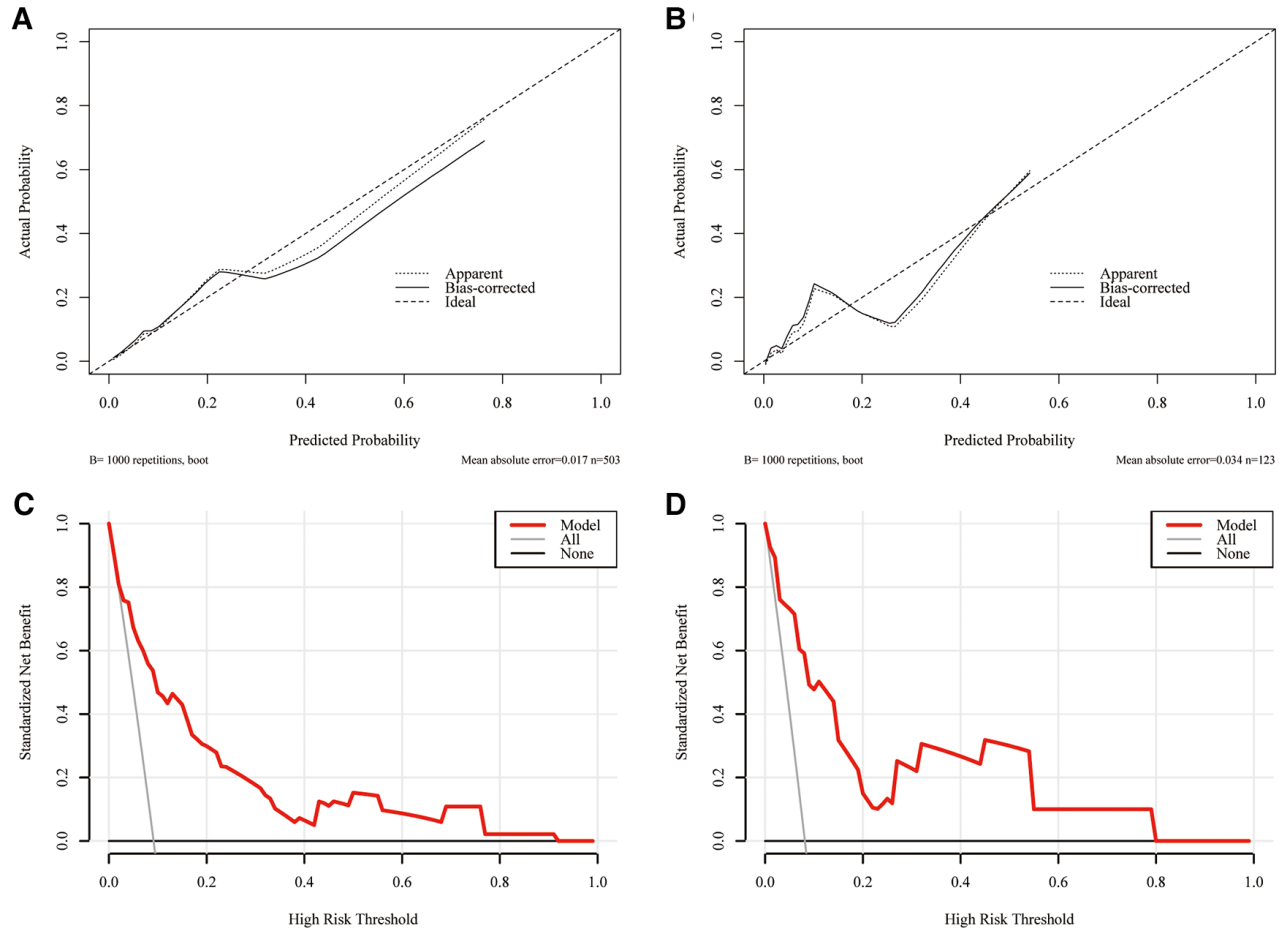
The calibration curves for comparing the predicted probabilities with actual probabilities and the DCA curves for estimating the net benefit of the prediction model. (**A**) The calibration curve in the training set; (**B**) the calibration curve in the validation set; (**C**) the DCA curve in the training set; (**D**) the DCA curve in the validation set.

A nomogram integrating all five significant factors was created. According to the analysis, capsular contact >50% was the most significant contributor to the prediction model, followed by ETE. The final scores were calculated by summing the total scores, and the risk rate of HVLNM was calculated (Figure 3). The calibration curves showed good agreement between the predicted and observed probabilities of HVLNM, with a mean absolute error of 0.017 and 0.034 (Figure 4). The DCA curves showed a threshold probability from 0.00 to 0.92 in the training set, suggesting a wide range of clinical utility (Figure 4).

## Discussion

Evidence has suggested that the prognosis of PTC was linked to the number and size of involved lymph nodes. Randolph et al. revealed a marked difference in the median risk of recurrence in pathological N1 patients between <5 positive nodes (4%, range 3%–8%) and >5 nodes (19%, range 7%–21%) (10). Moreover, an analysis based on data from the National Cancer Data Base and SEER database, showed that a higher number of metastatic lymph nodes (up to six metastatic) was associated with lower overall survival (HR 1.12, 95% CI 1.01–1.25). In contrast, no additional mortality risk was found with more positive nodes (HR 0.99, 95% CI, 0.99–1.05) (11). All these findings allude a more concerning message about HVLNM. Thus, the 2015 ATA guidelines have modified the clinical and pathological nodal status as a characteristic that can stratify the risk of recurrence in PTC patients. The status of more than five metastatic lymph nodes is classified as intermediate risk, indicating a >20% risk of recurrence (8).

Precise identification of patients with HVLNM preoperatively may facilitate the selection of rigorous screening and treatment protocols. However, the low diagnostic performance of lymph nodes in preoperative assessments is unfavorable for patients and physicians. It was reported that the diagnostic efficacy was unsatisfactory, with a pooled sensitivity between 0.31 and 0.35 for detecting involved lymph nodes in the central neck (22). Another meta-analysis reported that US + Computed Tomography and FNA cytology + FNA-thyroglobulin (FNA-Tg) showed good diagnostic performance (23). The limitations are that these methods are radiative or invasive. For these reasons, it would be helpful to establish a prediction model for PTC patients combined with demographic features and nodule characteristics using US.

In a previous study, preoperatively sonographic characteristics combined with serum Tg antibodies levels were found to help predict central LNM (CLNM) (24). Differently, our current study developed a preoperative nomogram based on preoperative sonographic patterns to visualize the prediction model for HVLNM in solitary CVPTC patients. The AUC was 0.842 in the training set and 0.875 in the validation set. Moreover, the HVLNM rate was found in 9.1% (46/503 cases) in the training set and 8.1% (10/123 cases) in the validation set, which was lower than that reported by Liu et al. (12.3%, 254/2,073 cases) (16). Different designs of the single lesion in this study, the inclusion criteria, or the sample capacity might have caused the divergence. When comparing the two groups in the training set, the data revealed that male sex, tumor size >10 mm, ETE on neck US, and capsular contact >50% were independent risk factors for HVLNM. Contrastingly, middle age and old age were significant protective factors. It is suggested that increased vigilance would be required for potential HVLNM patients.

There is no consensus regarding the impact of age on LNM, and age classification varies in different studies. Ito et al. pointed out that tumor progression was the greatest in young patients and the poorest in older patients with papillary thyroid microcarcinoma (PTMC), indicating that tumors presented increased aggressiveness in younger patients compared to older patients (25). A meta-analysis, including 9,369 PTC patients, reported that age <45 years increased the risk of lymph node metastasis (pooled OR 1.52, 95% CI 1.14–2.01, *P* < 0.00001) (26). In the study, age classification was consistent with that of two recent studies by Oh et al. and Shen et al. (19, 20). According to our data in the training set, people of middle age and older people groups had less than half the incidence of HVLNM compared to the young age group. Multivariate analysis revealed that middle-aged and older patients had lower rates of HVLNM than younger patients. In addition, studies have investigated age's influence on HVLNM in patients with PTMC and derived some approximations. For instance, Zhang et al. found that the risk of HVLNM in PTMC was significantly lower in patients of middle age (40–59 years) (OR 0.313, 95% CI 0.191–0.515) and older (≥60 years) patients (OR 0.085, 95% CI 0.012–0.633) (18). Another finding worth mentioning is that Liu et al. revealed that age ≥40 years was an independent protective factor (17). Similarly, elderly age (≥55 years) was also found to be a protective factor of high-volume CLNM within PTMC in Wei’s study (27). However, age was not found to be significantly different among participants in a study of 2,073 patients with PTC using the cutoff of 55 years (16). Overall, grouping by age varied in some studies; thus, detailed and well-recognized stratification of age groups may provide a more accurate risk assessment. Apart from age, our results demonstrated that the risk of HVLNM in males was 3.396 times higher than that in females (95% CI 1.579–7.304), indicating a similar adverse effect of male sex on LNM in previous studies (26).

Importantly, primary tumor size is the most intuitive parameter in the preoperative US that has long been analyzed in previous studies and included in several scoring schemes (AGES, AMES, and MACIS) (28–30). To distinguish PTMC and conventional papillary thyroid cancer, 10 mm is used as a benchmark. It has been proposed that tumors with a diameter larger than 10 mm have a higher incidence of invasion, LNM, and CLNM, thus requiring more radical tactics to improve outcomes (26, 31–33). Likewise, in our study, the HVLNM portion of patients with tumor size ≤10 mm was 4.2%, while in the >10 mm group was 18.8% in the training set. Multivariate analysis also demonstrated that a tumor diameter >10 mm significantly increased the risk of HVLNM.

Numerous studies have shown that ETE was recognized as a predictor of metastatic diseases, such as locoregional LNM and distant metastasis (34, 35). In addition, Feng et al. used the SEER database and single-center data to evaluate the correlation between demographic and clinicopathologic characteristics and CLNM in CVPTC patients (33). ETE on pathology was significantly different between the two groups (33). On the contrary, Tao et al. did not find significant associations of ETE with CLNM and lateral LNM (LLNM) in PTMC (36). However, the definition of ETE differed in some of these studies, and most were based on postoperative pathological results, which limited their utility in the presurgical setting. Lamartina et al. reported that by combining US signs of minimal or gross ETE and taking the presence of microscopic or gross ETE as a reference on histology, preoperative US achieved an accuracy of 81.5% (37). In addition, gross ETE is believed to have a higher incidence of tumor recurrence than microscopic ETE (38, 39). Accordingly, preoperative US signs of ETE are of great diagnostic importance. In our current study, the incidences of HVLNM were significantly different in terms of ETE. Nevertheless, further studies are still needed to determine the diagnostic criteria for ETE using US.

Few studies have estimated the degree of capsular contact and analyzed its impact on LNM in PTC (40–42). Animal and human studies suggested that lymphatic networks and vessels appeared denser at the periphery of the gland (43, 44). A study in Japan revealed that increased lymphatic density was also correlated with vascular endothelial growth factor-D expression and LNM in PTC patients (45). These findings could explain that the state of contact with the glandular capsule has facilitated the spread of the tumor to regional lymph nodes. Research in clinical practice concurred with these findings. Ye et al. found that capsular extension >50% was associated with LLNM in PTC (40). Kwak et al. found >25% contact with the adjacent capsule was a risk factor for LLNM in PTMC (46). Contrastingly. Lin et al. and Zeng et al. found no significant association between capsular contact and LLNM after multivariate logistic regression (47, 48). Different from previous studies, our study highlighted the association between capsular contact and HVLNM and found that capsular contact >50% was the most common in the HVLNM group in the training set, contributing the most to the prediction model. Since the all-around measurement is needed during the examination, real-time observation of US or dynamic images after the examination could benefit the analysis after the examination.

Despite the promising results, limitations remain in the current study. First, this was a retrospective case-control study at a single center, and selection bias was inevitable. Second, only a small number of patients had HVLNM, and comparisons were only made between the HVLNM and non-HVLNM groups, while factors related to other nodal statuses were not investigated. Moreover, our nomogram included only five factors, and potential variables might need to be analyzed and validated. Thus, a large-sample cohort study involving external validation from a multicenter study is required.

In conclusion, HVLNM is relatively uncommon in CVPTC patients with a solitary lesion. Male sex, larger tumor size (>10 mm), ETE on US, and capsular contact >50% increased the risk of HVLNM, whereas middle and old age were significant protective factors. These findings may be essential for implementing more vigilant and aggressive preoperative examinations and treatment strategies for CVPTC patients with a high risk of HVLNM based on the nomogram. Additionally, real-time US plays a vital role in preoperative assessment.

## Data Availability

The raw data supporting the conclusions of this article will be made available by the authors, without undue reservation.
